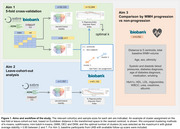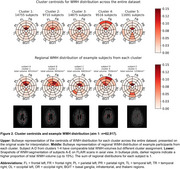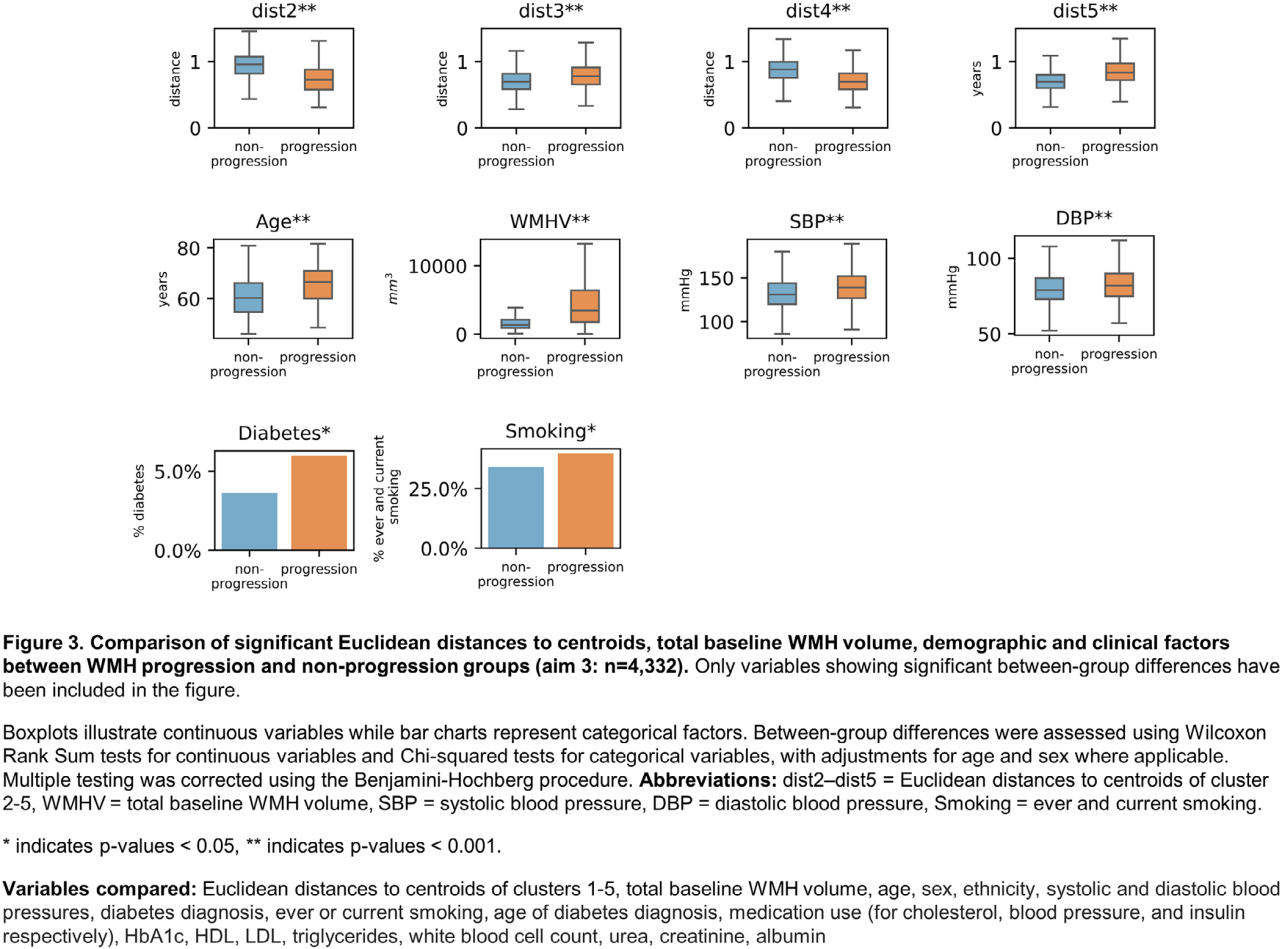# Location patterns of white matter hyperintensities in a multi‐cohort study — generalisability, evaluation, and longitudinal progression

**DOI:** 10.1002/alz70856_099916

**Published:** 2025-12-24

**Authors:** Xin Zhao, Ian B. Malone, David M Cash, Andrew Wong, Nishi Chaturvedi, Alun D Hughes, Jonathan M Schott, Jo Barnes, Carole H Sudre

**Affiliations:** ^1^ MRC Unit for Lifelong Health and Ageing, Department of Population Science and Experimental Medicine, University College London, London, United Kingdom; ^2^ Department of Medical Physics and Biomedical Engineering, University College London, London, United Kingdom; ^3^ Dementia Research Centre, UCL Queen Square Institute of Neurology, University College London, London, United Kingdom; ^4^ Hawkes Institute, Department of Computer Science, University College London, London, London, United Kingdom; ^5^ Institute of Cardiovascular Science, University College London, London, United Kingdom; ^6^ Hawkes Institute, University College London, London, United Kingdom; ^7^ Unit for Lifelong Health and Ageing, Department of Population Science and Experimental Medicine, University College London, London, United Kingdom

## Abstract

**Background:**

White matter hyperintensities (WMH) of presumed vascular origin are a neuroimaging manifestation associated with ageing, dementia, stroke and cognitive decline. Location‐based approaches are increasingly important for understanding WMH progression. This study aims to: 1) identify robust WMH spatial patterns, 2) validate their generalisability, and 3) assess their longitudinal relevance.

**Method:**

We analysed 62,917 participants from ADNI3 (*n* = 540), Insight46 (*n* = 462), SABRE (*n* = 765), and UK Biobank (UKB) (*n* = 61,150), aged 45‐97, with T1‐weighted and T2‐weighted FLAIR scans. WMH lesions were quantified using the BaMoS algorithm into 36 regional WMH volumes, scaled to intracranial volume (ICV) relative to the median ICV. Relative WMH distribution was derived as each regional volume divided by total WMH volume. The workflow is illustrated in Figure 1. Clustering was performed on square‐root‐transformed, normalised regional WMH markers (aim 1: *n* = 62,917). The optimal method and number of clusters k were determined using stability estimation (Yu et al. 2019) (20 bootstrap runs for k between 2 and 7, with 5‐fold cross‐validation). Jaccard‐based stability was computed at subject, cluster, and global levels. Clusters were validated using a leave‐cohort‐out approach in UKB (aim 2: training *n* = 1,767, test *n* = 61,150). Euclidean distances to cluster centroids, total WMH volume and 19 risk factors were compared between WMH progression and non‐progression groups (cut‐off at 250mm3/year) in UKB, adjusted for age and sex (Benjamini‐Hochberg corrected)(aim 3: *n* = 4,332).

**Result:**

SubKmeans with k=5 was optimal, achieving stability of 0.941±0.009 on training folds. Validation folds showed strong cluster reproducibility of 97.6%±1.0% and adjusted Rand index of 0.941±0.025. Leave‐cohort‐out validation showed moderate centroid shifts (stability 0.898, reproducibility 68.5% and adjusted Rand index 0.452). Centroids from primary and leave‐cohort‐out analyses showed strong alignment (Figure 2). WMH distribution was more diffuse in clusters 2 and 4 and more periventricular in clusters 1 and 3. WMH progressors were closer to centroids of clusters 2 and 4, further from clusters 3 and 5, older, had higher blood pressure (*p* <0.001), and were more likely to have diabetes and be ex‐ or current smokers (*p* <0.05) (Figure 3).

**Conclusion:**

This study identified five WMH distribution subtypes in a multi‐cohort setting, demonstrated their longitudinal relevance for WMH progression, supporting future location‐based analyses.